# Spatiotemporal dynamics of Spc105 regulates the assembly of the *Drosophila* kinetochore

**DOI:** 10.1098/rsob.110032

**Published:** 2012-02

**Authors:** Zsolt Venkei, Marcin R. Przewloka, Yaseen Ladak, Shahad Albadri, Alex Sossick, Gabor Juhasz, Béla Novák, David M. Glover

**Affiliations:** 1 Department of Genetics, University of Cambridge, Cambridge CB2 3EH, UK; 2 Wellcome Trust/Cancer Research UK Gurdon Institute, Cambridge CB2 1QN, UK; 3 Oxford Centre for Integrative Systems Biology, Department of Biochemistry, University of Oxford, Oxford OX1 3QU, UK; 4 Department of Anatomy, Cell and Developmental Biology, Eotvos Lorand University, Budapest 1117, Hungary

**Keywords:** centromere, chromosomes, KMN network, Mis12 complex, mitosis

## Abstract

The formation of kinetochores shortly before each cell division is a prerequisite for proper chromosome segregation. The synchronous mitoses of *Drosophila* syncytial embryos have provided an ideal *in vivo* system to follow kinetochore assembly kinetics and so address the question of how kinetochore formation is regulated. We found that the nuclear exclusion of the Spc105/KNL1 protein during interphase prevents precocious assembly of the Mis12 complex. The nuclear import of Spc105 in early prophase and its immediate association with the Mis12 complex on centromeres are thus the first steps in kinetochore assembly. The cumulative kinetochore levels of Spc105 and Mis12 complex then determine the rate of Ndc80 complex recruitment commencing only after nuclear envelope breakdown. The carboxy-terminal part of Spc105 directs its nuclear import and is sufficient for the assembly of all core kinetochore components and CENP-C, when localized ectopically to centrosomes. Super-resolution microscopy shows that carboxy-terminus of Spc105 lies at the junction of the Mis12 and Ndc80 complexes on stretched kinetochores. Our study thus indicates that physical accessibility of kinetochore components plays a crucial role in the regulation of *Drosophila* kinetochore assembly and leads us to a model in which Spc105 is a licensing factor for its onset.

## 2. Introduction

The kinetochore is essential for chromosome segregation and has been highly conserved in structure, despite the divergence of its subunits in primary sequence [1–4]. The structural core of the kinetochore has become known as the KMN network after its constituents: the **K**NL-1/Blinkin/Spc105 protein; the **M**is12/Mtw1/MIND complex and the **N**dc80/HEC1 complex (reviewed by Przewloka & Glover [5]); for uniformity, we will refer them here as Spc105, the Mis12 complex and the Ndc80 complex. In addition to acting as a molecular link between the centromere and microtubules, the KMN network also acts as platform for the kinetochore recruitment of proteins involved in the spindle assembly checkpoint, and in regulating microtubule capturing and plus end dynamics [5,6].

The details of interactions between KMN components differ in different organisms, but the common features may be exemplified in human cells. Here, the four subunits of the Mis12 complex are arranged linearly in a rod-shaped complex in the order Nnf1, Mis12, Dsn1 and Nsl1 [4]. One end of the complex interacts directly with the centromere [7,8], whereas the other forms a docking platform both for the Ndc80 complex and Spc105. The most distal molecule, Nsl1, interacts via distinct interfaces with the Ndc80 complex and with Spc105 [4]. All subunits of the Mis12 complex localize to the centromere from G2 until early G1, and the centromeric localization of these subunits is interdependent [9]. The heterotetrameric Ndc80 complex comprises Ndc80, Nuf2, Spc24 and Spc25, and has a long coiled-coil structure with globular domains at the two ends. The head domains of Spc24 and Spc25 interact directly with the Mis12 complex [10]. The tightly interacting Nuf2 and Ndc80 calponin-homology domains and the unstructured N-terminal tail of Ndc80 form the microtubule-binding interface [11]. Aurora B-dependent phosphorylation of the Ndc80 head domain regulates its microtubule-binding capacity as part of the error correction machinery. Spc105 provides a secondary microtubule-binding site of the KMN network. In human cells, its C-terminal part interacts with the Mis12 complex [4], and the amino-terminal part is responsible for binding Bub1 and BubR1 [12] and PP1 phosphatase [13].

The differences in arrangements and recruitment of KMN components in different organisms are of interest as they offer a route towards understanding the basic principles of kinetochore function. The recruitment hierarchy of the KMN network in *Caenorhabditis elegans* and *Drosophila melanogaster* differs from human cells. In *C. elegans,* RNAi studies show that recruitment of the Ndc80 complex depends on Spc105, and Mis12 complex recruitment is interdependent with Spc105 [14]. The assembly of the Mis12 complex with Ndc80 *in vitro* also requires Spc105 [15]. In *Drosophila,* studies of mutants and RNAi-treated cells have suggested a similar recruitment hierarchy of the KMN network [16,17]. There are also differences in the timing of centromeric recruitment of Mis12 complex subunits between *Drosophila* and human cells. The human Mis12 complex subunits recruit together to the centromere in late G2 and dissociate in early G1 [9]. Whereas *Drosophila* Mis12 protein is a constant element of the centromere, another subunit of the complex, Nsl1, recruits only in mitosis [18]. Accordingly, Mis12 appears upstream to Nsl1 in the recruitment hierarchy in *Drosophila* cells [18], in contrast to the interdependency of these molecules in human cells. Finally, the *Drosophila* Mis12 complex may differ significantly from the complex in other organisms because despite an extensive search, a Dsn1 orthologue has never been detected [19].

Interestingly, practically the entire human KMN network may be reconstituted *in vitro* from bacterially expressed subunits [8]. This suggests that the role of post-translational modifications may not be crucial in the process of the core kinetochore assembly (see also [20]) and that other mechanisms could directly regulate assembly of the KMN network. The data we present here accord with this notion.

We began this study to determine whether Spc105 might contribute to Mis12 function as we have previously hypothesized [19]. We show, by reassessment of the dependency relationships for kinetochore recruitment and by analysing the kinetics of KMN member recruitment, that assembly of the Mis12 complex at the kinetochore is dependent on Spc105 and is triggered by its nuclear import during prophase. The Ndc80 complex, on the other hand, can only be recruited following nuclear envelope breakdown (NEB). We find that the C-terminal part of Spc105 is not eliminated from the interphase nucleus, where it associates together with all Mis12 complex members to the centromere. Indeed, a C-terminal fragment of Spc105 is able to promote assembly of all Mis12 complex components on ectopic sites during mitosis. This is supported by our high-resolution mapping of this part of Spc105 to the proximity of Mis12 complex components, Mis12 and Nsl1, and the Ndc80 complex component Spc25/Mitch. Together our studies point to the central role of Spc105 in *Drosophila* kinetochore assembly and to the importance of controlling the accessibility of individual components to regulate assembly.

## 3. Results

### 3.1. Mitotic Mis12 complex is assembled at prophase concomitant with Spc105 nuclear import

**Figure 1.**
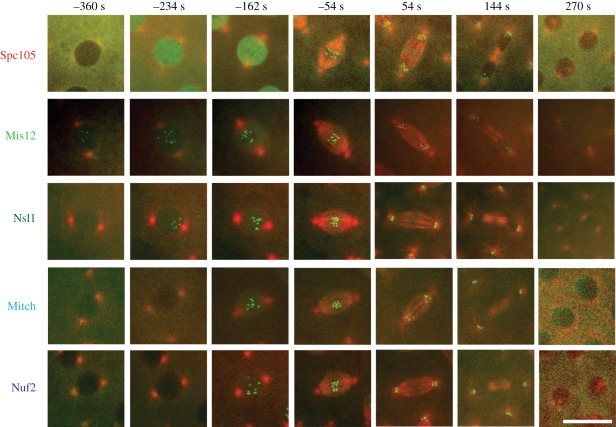
Recruitment of core kinetochore assembly throughout nuclear division cycle 12 in the syncytial embryo. Selected time frames from interphase 12 to interphase 13, presenting single nuclei from embryos with different EGFP fusions (EGFP in green and tubulin in red). In interphase (−360 s), there are only Mis12::EGFP proteins detectable at the centromere. Spc105 is clearly excluded from the nucleus. In early prophase (−234 s), Spc105 import is in progress, and Mis12 and Nsl1 accumulate at the kinetochore. Mitch and Nuf2 recruit later.

**Figure 2.**
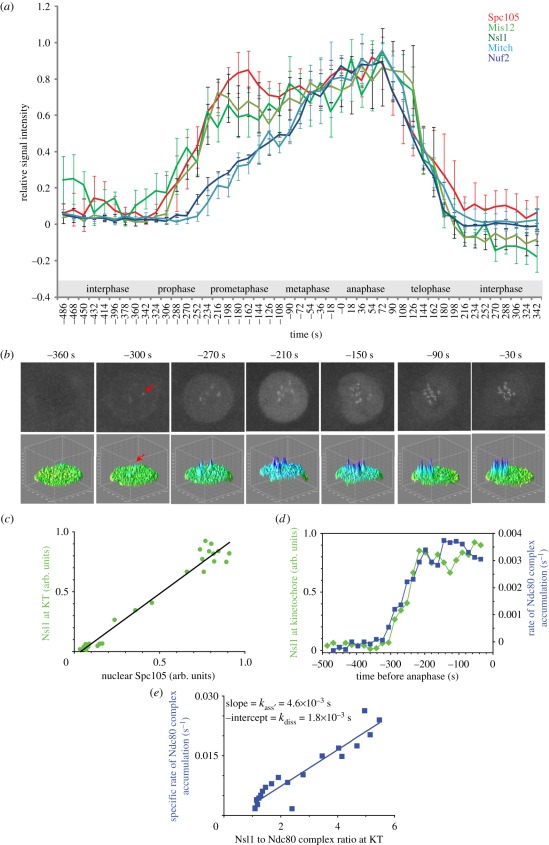
Quantitative analysis of recruitment of core kinetochore components throughout nuclear division cycle 12 in the syncytial embryo. (*a*) Quantitative comparison of kinetochore recruitment of KMN network proteins. For each protein, the average of maximal intensity values of kinetochore signals (3–3 nuclei from two embryos) are plotted as a function of time. Error bars represent standard deviations. The first time frame after AO was defined as zero time point to align data. (*b*) Exemplary still images from the electronic supplementary material, video S3, which illustrate that Spc105-GFP is recruited to centromeres immediately after its import into nuclei during prophase. Each cell image is accompanied by the surface intensity plot of the nucleus area. Red arrows point at the first detectable centromeric signal. (*c*) Nsl1 levels at the kinetochore in arbitrary units as a function of nuclear Spc105 levels (see text and electronic supplementary material, supplementary kinetic analysis). (*d*) Accumulation of Nsl1 level and the rate of accumulation of the Ndc80 complex at the kinetochore plotted against time in relation to AO. (*e*) Rate of Ndc80 complex accumulation plotted as a function of the Nsl1 : Ndc80 complex ratio at the kinetochore (see text and electronic supplementary material, supplementary kinetic analysis)

Kinetochore assembly is a defining aspect of mitotic progression and yet the exact sequence of events whereby the kinetochore is assembled upon entry into mitosis is poorly characterized. Knowledge of the precise timing and kinetics of recruitment of individual KMN components to centromeres would greatly contribute to the understanding of mechanisms of kinetochore assembly and may identify key steps in coordinating this process. In searching for a system that would give sufficient resolution of the sequence of these events, we turned to the *Drosophila* syncytial embryo, which shows synchronous cycles of mitoses that are extremely similar in length from one embryo to another. We generated transgenic flies that would express the KMN components, Mis12, Nsl1, Mitch/Spc25, Nuf2 or Spc105, each tagged with green fluorescent protein (GFP), and filmed the behaviour of these kinetochore components in the synchronous syncytial nuclear division cycles (electronic supplementary material, videos S1 and S2; [18]). We then analysed their kinetochore recruitment in successive time frames of single nuclei in cycle 12 (figure 1; electronic supplementary material, figure S1). The time between NEB (monitored by tubulin accumulation in the nucleus) and anaphase onset AO (set as zero time; electronic supplementary material, figure S2*a*,*b*) was highly reproducible between individual nuclei and between different embryos, as previously published [21]. We did not detect any toxicity or any mitotic defects associated with expression of the GFP-tagged KMN component in any of these lines with the exception of Spc105. In this case, 15 per cent of embryos expressing 3.5-fold elevated levels of GFP-tagged Spc105 displayed collapsing spindles in these late cycles. To assess association of each KMN component with the kinetochore, we measured the GFP signal intensity in the nucleus or around the chromosomes in each time frame (electronic supplementary material, figures S1 and S2*c*) and normalized the resulting values (see methods and legends of electronic supplementary material,  figure S2*d*,*e*). The final data plot represents mean values obtained from three nuclei from each of two embryos from two different lines (electronic supplementary material, figure S2*f*–*h*). Using this approach, we analysed the timing of recruitment of representative KMN network components, Spc105, Mis12, Nsl1, Mitch and Nuf2, expressed as enhanced green fluorescent protein (EGFP) fusions. The cumulative data are shown in figure 2*a*.

In interphase, Mis12-GFP showed a weak punctuate signal and was otherwise equally diffuse in the nucleus and cytoplasm. A dramatic increase in accumulation of both Mis12-GFP and Nsl1-GFP on the nascent kinetochore begins at the same time frame in early prophase, and the kinetics of kinetochore association subsequently overlapped. The Ndc80 complex components, Mitch and Nuf2, were excluded from the nucleus in interphase. In contrast, their recruitment began only after NEB and lagged behind the Mis12 complex. The two Ndc80 complex components were, however, recruited with similar kinetics. Spc105 followed a distinct pattern. It was clearly excluded from the nucleus at interphase and imported into the nucleus prior to NEB. When we observed Spc105-GFP fluorescence at focal planes at which it was maximal, this revealed the protein to associate with kinetochores instantaneously upon nuclear import (an arrow points at the first kinetochore signal in figure 2*b*; see also electronic supplementary material, figure S2*i* and video S3). We found that the recruitment of the Mis12 complex components (e.g. Nsl1) to the kinetochore followed very similar kinetics to accumulation of Spc105-GFP into the entire nucleus (figure 2*c*).

These data suggested codependent recruitment and assembly of the mitotic Mis12 complex and Spc105 com encing at Spc105 nuclear import, followed by Mis12/Spc105-dependent kinetochore recruitment of the Ndc80 complex commencing at NEB. We analysed these inter-relationships by applying a differential equation relating the reversible binding of one molecule of interest to another already at the kinetochore (see electronic supplementary material, supplementary kinetic analysis: equation (1)). In this analysis, we first considered the association of Spc105 with Mis12 complex formed at the centromere. By plotting the level of the Mis12 complex component, Nsl1, as a function of Spc105 levels, we found a linear relationship with the slope and the intercept of a regression line not significantly different from one and zero. According to electronic supplementary material, equation (2) (see electronic supplementary material), this relationship indicates rapid and strong (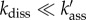) binding between the two partners, and therefore Spc105 level at the kinetochore is proportional to the total amount of Mis12 associated with the centromeric binding site. In contrast, Ndc80 complex level (assessed as the mean value for its two equimolar components, Nuf2 and Spc25/Mitch) at kinetochores does not show linear correlation with Mis12 complex (not shown). However, when we calculated the rate of accumulation of Ndc80 complex at the kinetochore (the slope of the blue curves from figure 2*a*) and plotted together with the level of Nsl1 as a function of time, we found a strong correlation (figure 2*d*). Slow binding of Ndc80 complex relative to the change in total Mis12 complex at the nascent kinetochore may be described by equation (3) (see electronic supplementary material). By plotting the specific rate of Ndc80 accumulation as a function of the Mis12 : Ndc80 ratio (after rearranging equation (3) into equation (4); see electronic supplementary material), we obtained a straight line with a slope and a negative intercept corresponding to 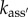 and *k*_diss_ values of 4.6×10^–3^ and 1.8×10^–3^ s^–1^, respectively (figure 2*e*). Thus, these considerations of the relationships between the kinetics of recruitment of the three main components of the KMN network would accord with the codependency of the Mis12 complex and Spc105 for their recruitment (which is shown below) in order to provide a structure upon which the Ndc80 complex can be subsequently recruited.

### 3.2. Recruitment of Mis12 complex to nascent kinetochore at mitotic entry depends on Spc105

The kinetics of kinetochore assembly and the spatio-temporal relationships between the KMN components upon entry into and progression through mitosis led us to reassess the recruitment dependencies of the different KMN components upon each other and to ask whether these changed upon mitotic entry. As the kinetic analysis suggested the particular importance of the recruitment of the Mis12 complex and the nuclear import of Spc105, we focused upon re-examining the recruitment dependencies of these components. In so doing, we chose to use antibodies to localize endogenous KMN molecules [18,22] in order to avoid potential artefacts that might occur when studying the localization of EGFP-tagged proteins.

**Figure 3.**
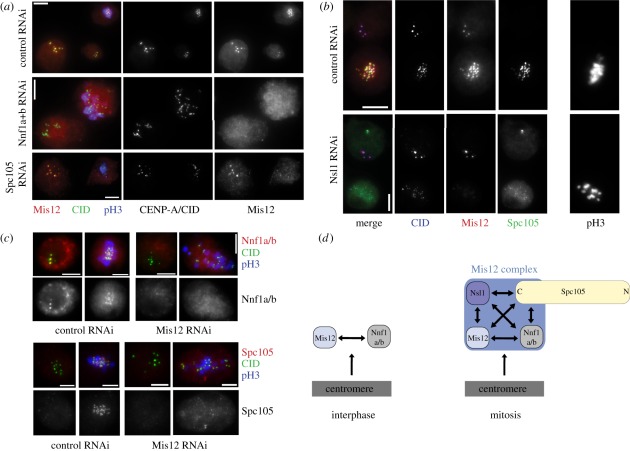
Recruitment dependencies in the core kinetochore during interphase and mitosis. Exemplary results from RNAi-based experiments. The localization of proteins was studied by immunolocalization with specific antibodies. Anti-phospho(Ser10) histone H3 antibody (shown as ‘pH3’) is a marker for mitotic cells. (*a*) Centromeric/kinetochore localization of Mis12 protein depends on Nnf1a/b in both interphase and in mitotic cells, but only its mitotic localization is dependent on the presence of Spc105. (*b*) Nsl1 affects the mitotic localization of Mis12 and Spc105 at kinetochores, but not the centromeric localization of Mis12 during interphase. (*c*) Both Nnf1a/b and Spc105 depend on Mis12 for their localization to centromeres. (*d*) Diagram shows summary of recruitment dependency results. Interphase localization of Mis12 and Nnf1a/b is mutually dependent, but independent of Nsl1 and Spc105. Mitotic localization of all Mis12 complex components and Spc105 is interdependent. Scale bars, 5 µm.

We found that Mis12 and Nnf1a/b gave clear signals on centromeres during interphase [18] (see also electronic supplementary material, figure S3), although their intensities varied widely from cell to cell. Knockdown of either Mis12 or Nnf1a/b by RNAi revealed that they were mutually dependent for their interphase localization (phospho-histone H3 negative cells, shown in figure 3*a*). Nsl1 or Spc105 are not present at centromeres during interphase [18] (electronic supplementary material, figure S3) and accordingly their knockdown did not reduce signal intensities of Nnf1a/b and Mis12 on centromeres at this stage of the cycle (figure 3*a*). We therefore concluded that the interphase recruitment of Mis12 and Nnf1a/b is reciprocally dependent, but independent of Nsl1 and Spc105.

The immunostaining of fixed untreated D-mel2 cells revealed robust localization of Mis12, Nsl1 and Spc105 on mitotic kinetochores [18,22] (electronic supplementary material, figure S3). Surprisingly, the immunostaining of Nnf1a/b at kinetochores was weaker than its centromeric staining in interphase (electronic supplementary material, figure S3). As Nnf1a/b is believed to be an equimolar part of the stoichiometric Mis12 complex in mitosis, it seems likely that this diminished staining is due to epitope masking upon complex assembly rather than loss of the protein from the kinetochore. Interestingly, we found that the mitotic recruitment of Mis12, Nnf1a/b, Nsl1 and Spc105 showed complete interdependence (phospho-histone H3 positive cells in figure 3*a*–*c*, summarized in figure 3*d*). We also found a strong pair-wise interdependency between Mis12 and Nnf1a/b (figure 3*a*,*c*), and between Spc105 and Nsl1 (figure 3*b*), for kinetochore recruitment. Not only was the Mis12-Nnf1a/b pair required for the localization of Nsl1-Spc105, but Mis12 and Nnf1a/b were not stably associated with the kinetochore in the absence of Nsl1 and Spc105. Thus, the centromeric presence of Mis12 and Nnf1a/b is Nsl1- and Spc105-independent in interphase, but Nsl1- and Spc105-dependent during mitosis (figure 3*d*). This remarkable change in the recruitment dependency suggests the existence of a regulatory switch operating at mitotic entry (see §4).

### 3.3. Exclusion of Spc105 from the interphase nucleus prevents premature initiation of kinetochore assembly

**Figure 4.**
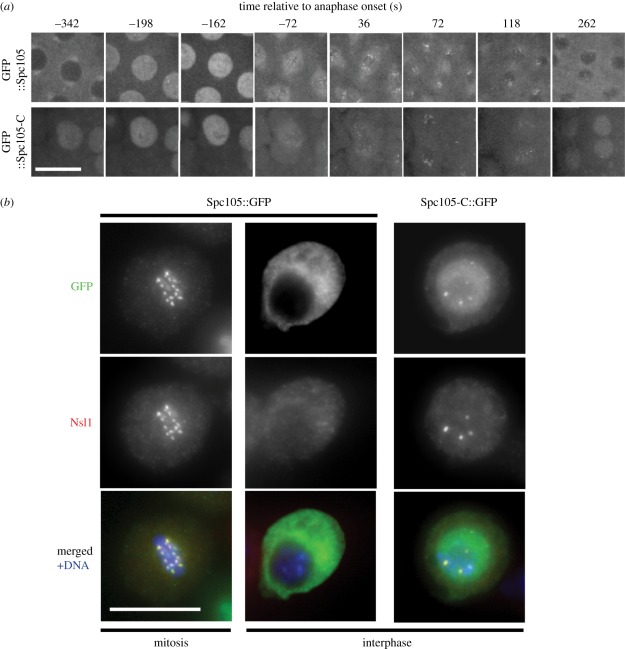
Nuclear exclusion of Spc105, and initiation of Mis12 complex assembly in interphase by Spc105 kinetochore-binding domain. (*a*) Sub-cellular localization of EGFP::Spc105 and EGFP::Spc105-C throughout a cortical cleavage division. Full-length protein is excluded from the nucleus in interphase and accumulates in the nucleus in prophase. After mitosis, Spc105 is excluded from the nucleus. Spc105-C is not excluded from the nucleus in interphase but prophase accumulation is comparable with full-length protein. Scale bar 10 µm. (*b*) Recruitment of Spc105-C and Nsl1 to the interphase centromere. Nuclear import of Spc105-C in interphase is sufficient to recruit itself and Nsl1 to the centromere. This capacity of Spc105-C depends on the functionality of its nuclear localization signal. Scale bars, 10 µm.

The Spc105 protein appears exceptional among the *Drosophila* KMN proteins in being clearly excluded from the nucleus in interphase and specifically imported during prophase. This shuttling of the protein between cytoplasm and nucleoplasm occurs not only in syncytial embryos, but also in dividing cells at later stages of embryogenesis (electronic supplementary material, video S4). The similarity of the kinetics of nuclear import of Spc105 with the increased accumulation of Mis12 components at the kinetochore upon mitotic entry suggested that Spc105's nuclear import might be critical for assembly of the Mis12 complex at the kinetochore. To understand better the regulation of Spc105's localization, we divided the molecule into two parts and found that the C-terminal part localized to the nucleus when transiently expressed in culture D-Mel cells. We compared the nuclear export–import dynamics of the C-terminal, kinetochore-binding part (1173–1959aa and Spc105-C) with the full-length protein in the living syncytial embryo (figure 4*a*; electronic supplementary material, videos S5 and S6) and found that although its levels increased in prophase nuclei, Spc105-C was retained in the nucleus during interphase and not excluded like the full-length protein (figure 4*a*). Therefore, Spc105-C contains a sequence motif enabling prophase nuclear import, but lacks the motif required for its nuclear exclusion in interphase.

The expression of Spc105-C gave a strong dominant-negative phenotype; all embryos died in the later syncytial blastoderm stages and had irregular numbers of nuclei of divergent sizes at the cortex (electronic supplementary material, figure S4). The irregular spacing of nuclei resulted from aberrant figures falling away from the cortex, leaving behind free centrosomes, as has been described for other mitotic mutants at this stage. This dominant-negative phenotype is in accord with previous observations in eye discs, where, although Spc105-C is able to partially rescue the mitotic defects of a *spc105* mutant, a strong dominant phenotype was also reported [17]. A similar dominant phenotype is seen following expression of human Spc105-C in cultured cells [17,23].

Schittenhelm *et al*. [17] also showed that Spc105-C was able to bind the centromere in mitosis and directly interacts *in vitro* with Mis12 complex subunits. When we introduced Spc105-C into cultured D-mel2 cells by transient transfection, we found it became localized throughout the nucleus in interphase and was also present in discrete, stronger foci, which represent centromeres (figure 4*b*). This was in contrast to full-length Spc105, which was restricted to the cytoplasm of such cells. We then asked whether other kinetochore proteins might be recruited to the interphase centromere in the presence of Spc105-C. We found in cells expressing Spc105-C that Nsl1, normally never present on interphase centromeres [18], was recruited to punctuate foci that colocalized with CID/CENP-A (figure 4*b* and data not shown). We were unable to detect several other kinetochore-associated proteins (including Ndc80, Mitch, Rod, BubR1, Aurora-B, Polo and full-length Spc105) on the interphase centromere in the presence of Spc105-C (data not shown), and the intensity of interphase staining for the Mis12 and Nnf1a/b antigens appeared to be unchanged. Thus, Spc105-C is sufficient to recruit Nsl1 to join Mis12 and Nnf1a/b, but not other kinetochore proteins, at interphase centromeres.

### 3.4. Centrosomal localization of kinetochore-binding domain of Spc105 results in the recruitment of all KMN network components and CENP-C to ectopic sites

**Figure 5.**
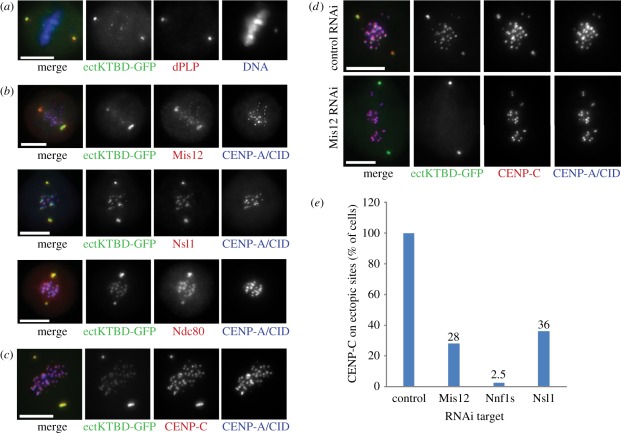
Ectopic expression of the kinetochore-binding domain of Spc105 fused with EGFP (ectKTBD-GFP). (*a*) ectKTBD-GFP (green) colocalizes with dPLP (red) in Dmel-2 cells. DNA counterstained with DAPI (blue). (*b*) KMN network components (red) Mis12, Nsl1 and Ndc80 are recruited to centrosomes in cells expressing ectKTBD-GFP (green). CENP-A/CID (blue) signals mark the endogenous centromeres; CENP-A/CID is not recruited to ectopic sites. (*c*) CENP-C (red) localizes to endogenous centromeres and to ectopic sites, just like ectKTBD-GFP (green). (*d*) Negative control after RNAi shows the pattern of staining identical to the one shown in (*c*). However, the depletion of Mis12 complex components (here Mis12 protein is given as an example) displaces ectKTBD-GFP from endogenous centromeres and CENP-C from ectopic sites. (*e*) After RNAi, described in (*d*), cells showing the ectopic CENP-C localization were counted (*n* = 40 in each case). All ectKTBD-GFP expressing cells show ectopic CENP-C in a control knock down, but CENP-C is retained on centrosomes only in a fraction of cells after depletion of the individual Mis12 complex subunits. Scale bars, 10 µm.

The above findings suggested that Spc105 may play a central role in the assembly process and that it might be able to promote assembly of the complex if targeted to an ectopic site. We have previously shown that it is possible to recruit all components of the KMN network to the centrosome by targeting CENP-C to this site [22]. To assess the importance of Spc105 in kinetochore assembly, we employed the same strategy and targeted a fragment of the protein to centrosomes. This fragment comprised the C-terminal 394 amino acids of Spc105 (aa 1566–1959), which we term the kinetochore-binding domain (KTBD), fused with EGFP on its amino terminus and the Plk4 centrosome-targeting domain on its carboxy-terminus. We established stably transformed Dmel-2 cell lines in which this construct (ectKTBD-GFP) could be inducibly expressed. Following induction, the fusion protein became colocalized with the centrosomal marker, dPLP (figure 5*a*). All the KMN network components that we tested were also recruited to these ectopic sites (figure 5*b*). Thus, the binding domain of Spc105 itself is sufficient to coordinate assembly of the KMN network. Surprisingly, we also found that the centromeric protein, to which the Mis12 complex binds, CENP-C, was also recruited to ectopic sites in mitotic cells (figure 5*c*). This was unexpected, because CENP-C is thought to be deeply embedded in the nucleosomal structures. We hypothesized that the assembly of the KMN network, specifically Spc105 and the Mis12 complex, creates a molecular interface able to recruit CENP-C to the ectopic kinetochore complex. If this were the case, then knockdown of Mis12 complex components ought to disable ectopic CENP-C recruitment even though ectKTBD-GFP was still present at the centrosome. Indeed, we found that after depleting Mis12 (figure 5*d*), Nsl1 or both Nnf1 proteins by RNAi, the localization of CENP-C to endogenous centromeres was not affected, but its recruitment to ectopic sites was highly reduced (figure 5*e*). Additionally, as expected from our studies of recruitment dependencies, we found ectKTBD-GFP no longer localized to endogenous kinetochores after knockdown of Mis12 complex components, reflecting the codependence of Spc105 and the Mis12 complex subunits in kinetochore recruitment (figure 3).

In summary, the ectopic localization of the kinetochore-binding part of Spc105 is sufficient to artificially assemble the KMN network on centrosomes. Additionally, those ectopic kinetochores are capable of also recruiting CENP-C to centrosomes, most probably because of the high-binding affinity of Mis12 complex to CENP-C. These findings underscore the central importance of Spc105 in the assembly of *Drosophila* kinetochore.

### 3.5. C-terminal part of Spc105 is positioned at overlap between Mis12 and Ndc80 complexes in the assembled kinetochore

**Figure 6.**
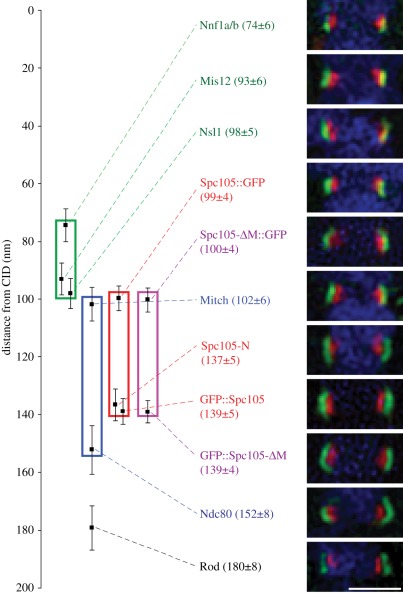
Architecture of the *Drosophila* core kinetochore. Bioriented sister kinetochore pairs in metaphase D-mel2 cells and in cells expressing different GFP-fusions. In single panels, seven different endogenous kinetochore epitopes and GFP fusions on both ends of Spc105 and Spc105-*Δ*M are highlighted in green, in combination with CID in red and DNA in blue. Numbers in parentheses are average distances from CID ± s.e.m. Green box represents Mis12 complex, red box Spc105, blue box Ndc80 complex. The scale is set equal to zero at position of CID. Average distances of single epitopes from CID are highlighted as black squares. *n* = 50, error bars represent s.e.m. Scale bar, 1 µm.

If the C-terminal part of Spc105 were to coordinate kinetochore assembly, we argued that it should occupy a position that would accord with such a role. We therefore mapped its exact position with respect to all other KMN components on kinetochores under tension. Although such spatial arrangements have been described for bioriented metaphase chromosomes of budding yeast, chicken and human cells [24–26], the existing measurements of the relative positions of *Drosophila* kinetochore components were carried out only on detached chromosomes [17,27]. We therefore localized *Drosophila* KMN components on bioriented kinetochore pairs at metaphase that were under tension by the criterion that their intercentromeric distance was greater than 800 nm [28]. We acquired three-dimensional image stacks of fixed D-mel2 cells stained to reveal these proteins using an Optical Microscope eXperimental (OMX) in structured illumination (SI) mode (see electronic supplementary material, methods and video S7). Wherever possible, we used immunostaining to localize endogenous kinetochore proteins relative to the inner centromeric protein CID/CENP-A (figure 6; see also electronic supplementary material, figure S5*a*–*d*).

**Figure 7.**
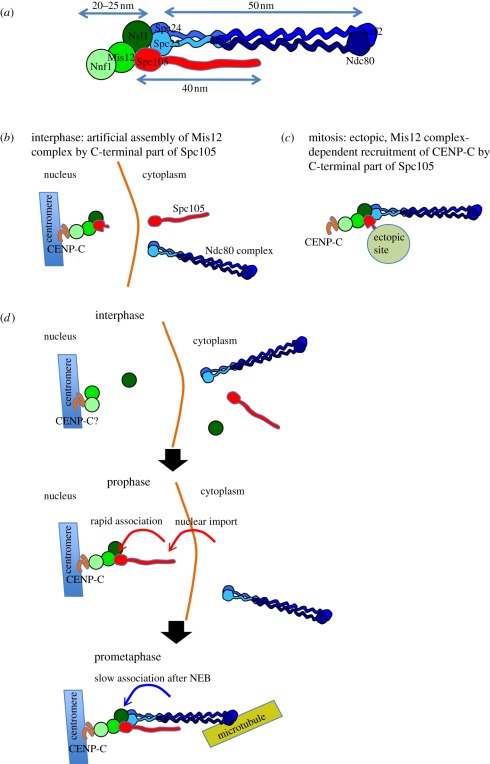
Key roles of Spc105 in the *Drosophila* KMN network. (*a*) Relative positions of the KMN components as a schematic of data from figure 6. (*b*) Schematic of the data presented in figure 4. The C-terminal fragment of Spc105 is not excluded from the nucleus at interphase and so can promote the association of Nsl1 with Mis12 and Nnf1 at the centromere. (*c*) Schematic of the data presented in figure 5. Ectopic localization of the kinetochore kinetochore-binding domain of Spc105 at the centrosome results in the recruitment of all KMN components and CENP-C to this ectopic site. Recruitment of CENP-C to the ectopic site is Mis12 complex-dependent and is dramatically reduced following RNAi of any of the Mis12 complex components. (*d*) Schematic of the assembly of the kinetochore to summarize our findings. In interphase, Mis12 and Nnf1a/b are associated with the centromere. In prophase, Spc105 is imported into the nucleus and rapidly associates with Mis12 complex members on the nascent kinetochore. Ndc80 complex is recruited after NEB, its rate of recruitment being proportional to the assembled Mis12 complex and Spc105 on the nascent kinetochore.

We found Nnf1a/b to lie closest to the centromere in accord with its interaction with the N-terminal part of the centromeric protein CENP-C [22]. Mis12 and Nsl1 were found 20–25 nm more distal in a cluster with the C-terminus of Spc105-C (tagged with GFP, see also below) and the Ndc80 complex subunit Spc25/Mitch (figures 6 and 7*a*). This clustering is reminiscent of human cells, where Nsl1 acts as an interface between Mis12 complex, the C-terminus of Spc105 and the head domains of Spc24-Spc25 in the Ndc80 complex [4]. It is entirely in accord with our above finding that the C-terminal part of *Drosophila* Spc105 could recruit all KMN components to an ectopic site. It is notable that the *Drosophila* Mis12 complex is similar in length to its human and yeast counterparts, which are proposed to form a 22–25-nm-long complex in which the components have the following linear order: Nnf1, Mis12, Dsn1, Nsl1 [1,2,4].

Our antibodies against the Ndc80 complex members, Spc25/Mitch and Ndc80, stained sites that were 50 nm apart (figures 6 and 7*a*; also strikingly similar to the 45 nm human Ndc80 measured in both Taxol-treated and untreated metaphase cells [26], and 57 nm when assembled *in vitro* [10]). It is somewhat longer than it is on unattached kinetochores, where it was reported to be 22 nm long [27]. This last measurement is likely to reflect relaxation of the complex upon detachment as it is similar to the value of 18 nm reported for the complex in nocodazole-treated HeLa cells [26].

Staining of Spc105 by our antibody, directed against its N-terminus, and by an N-terminal GFP tag, indicated this end of the molecule to lie 40 nm more distal than its C-terminus (figures 6 and 7*a*). There are no data on the length of human Spc105, but in budding yeast, the ends of Spc105 appear to lie 16 nm apart, consistent with this protein being less than half the size of its fly orthologue [24]. Under our conditions, *Drosophila* Spc105 seems to be in an extended conformation since, on detached chromosomes, the ends were only 9 nm apart [17]. This led us to ask whether stretching of Spc105 might contribute to the intrakinetochore stretch postulated to regulate spindle assembly checkpoint activity [26,28,29]. However, we found no difference in the distances between the two ends of Spc105 (electronic supplementary material, figure S5*e*–*h*) on bioriented chromosomes in the presence or absence of low concentrations of Taxol as used previously [28], although both ends were about 6 nm more distant from CID after Taxol treatment (electronic supplementary material, figure S5*e*–*h*). Finally, the deletion of a non-essential central region of Spc105 (amino acids 589–1268) had no effect upon the localization of the N- and C-termini of the molecule (figure 6), in accord with the ability of this deleted variant to rescue an *Spc105* mutant [17]. Together, these findings suggest that Spc105 occupies a key position at the interface between the Mis12 and Ndc80 complexes. Its functionality lies in its N- and C-terminal domains, the former lying alongside the central part of the Ndc80 complex and the latter lying close to Nsl1 and Mis12 in the Mis12 complex and Spc25/Mitch component of the Ndc80 complex.

## 4. Discussion

Several of the findings of our study highlight the importance of *Drosophila* Spc105 for kinetochore assembly. First, the timing of Spc105′s nuclear import is critical as this enables the onset of the assembly process. Second, recruitment dependencies indicate Spc105 is essential to assemble the Mis12 complex in mitosis, in agreement with previous findings [17] and supported by our present measurements of the kinetics of kinetochore association of these molecules. Third, the continued presence of the C-terminal part of Spc105 in the nucleus led to its accumulation together with all Mis12 components at the centromere (figure 7*b*), and this subsequently led to mitotic defects. Fourth, the importance of the C-terminal part of Spc105 in kinetochore assembly in mitosis is shown by its ability to recruit all KMN components and CENP-C, the centromeric-binding protein for the KMN, to an ectopic site (figure 7*c*). Finally, the position of the C-terminal part of Spc105 at a site where it could potentially interact with Nsl1 and Mis12 of the Mis12 complex and Mitch/Spc25 of the Ndc80 complex is in accord with its ability to recruit all components of the KMN complex to this ectopic site.

The unique ability of Spc105 to participate in nuclear transport appears key to the kinetochore assembly process. Spc105 is excluded from the nucleus in interphase and its import into the prophase nucleus provides the trigger for kinetochore assembly. The similar timing of this import to the import dynamics of both *Drosophila* and human Cyclin B1 [30,31] points to a direct link with the regulation of mitotic entry. Recent work demonstrated that the activation of Cyclin B1–Cdk1 immediately triggers its rapid accumulation in the nucleus through a 40-fold increase in nuclear import that remains dependent on Cdk1 activity until NEB [32]. Thus, this wave of Cyclin B–Cdk1-triggered nuclear import, bringing with it Spc105, would ensure the kinetochore is prepared for mitosis when NEB occurs. After nuclear import, Spc105 is incorporated immediately into the nascent kinetochore and the kinetics of its incorporation matches those of Mis12 components indicating that both rapidly associate. The Ndc80 complex is excluded from the nucleus at this stage even though its small subunits, Spc24 and Mitch/Spc25, are of a size that should be able to diffuse through nuclear pores. This suggests that the Ndc80 complex might be assembled in the cytoplasm only to be incorporated into the kinetochore upon NEB. Once the nascent kinetochore is accessible, the rate of association of Ndc80 complex is relatively slow and is proportional to the levels of the Mis12 complex and Spc105 already incorporated. Our data suggest that regulation of KMN network assembly may rely, at least partially, on the accessibility of individual components or subcomplexes rather than specific post-translational modifications. Such regulation also seems likely in vertebrates. The ability to assemble the entire human KMN network from subunits expressed in bacteria in the absence of post-translational modifications supports this notion [8].

Our findings that Mis12, Nnf1a/b, Nsl1 and Spc105 are interdependent for their mitotic recruitment to the nascent kinetochores of *Drosophila* (figure 3) suggest these four molecules interact in a coordinated manner. The four subunits of human Mis12 complex that include Dsn1 (but excluding Spc105) also show this interdependence [9]. The fact that the mitotic recruitment of Mis12 and Nnf1a/b depend on both Nsl1 and Spc105 would accord with the suggestion that Spc105 may substitute for the role of Dsn1 in the *Drosophila* Mis12 complex [19]. Although the Mis12 components, including Spc105, recruit interdependently, we see some stronger pair-wise interactions, for example, between Mis12 and Nnf1a/b, and between Nsl1 and Spc105. This is similar to the recently described behaviour of human and yeast Mis12 complex subunits [1,2,4]. Our reciprocal dependency experiments point to the central role of Spc105 in *Drosophila* that is similar to its counterparts in *C. elegans* [33] and *Schizosaccharomyces pombe* [34], and differs from human cells [4,23].

Of all the kinetochore components, Mis12 and Nnf1a/b are already associated with centromeres in interphase cells. At present, we do not know whether the interphase localization of Mis12 and Nnf1 occurs via their direct interaction with CENP-C (figure 7*d*). However, we show that their presence at the centromere is mutually dependent, suggesting they do interact but are unable to form the complete mitotic Mis12 complex because Nsl1 cannot be recruited and Spc105 is not present. The nature of the Mis12-Nnf1a/b interaction is thus likely to change in mitosis according to dependency of Mis12 and Nnf1a/b upon Nsl1 and Spc105 for their presence at the kinetochore. The indication of a similar change in organization between interphase and mitosis is seen for the human Mis12 protein, which shows highly dynamic association with the centromere in interphase but is stably associated with the mitotic kinetochore [9,35]. However, whereas in human cells Nsl1 is associated with interphase centromeres, where it can interact with HP1, we could neither see Nsl1 in the *Drosophila* interphase centromere [16,18] nor could we detect its direct interaction with HP1 (results from unpublished *in vitro* interaction assay experiments). This perhaps is not surprising, because *Drosophila* HP1 does not contain the C-terminal extension, which was shown to be responsible for the interaction between human Nsl1 and HP1 [4,36]. The change of recruitment interdependencies between interphase and mitosis was reported before by others [37], although in respect of other kinetochore proteins. Nevertheless, this previous study and our current findings point to the importance of the dynamics of structural changes in the sequential process of kinetochore assembly and function.

It is striking that despite the enormous divergence of their primary sequences, our charting of the dimensions and positions of the *Drosophila* KMN components show them to have extremely similar spatial relationships relative to the inner centromeric protein, CID/CENP-A (figure 7*a*), as their human counterparts, when kinetochores are attached to microtubules and under tension [24,26]. This similarity of spatial organization is even more striking considering that, unlike human cells, a fly orthologue of the Mis12 complex component, Dsn1, has never been identified [19]. Yet the physical distance between Nnf1a/b and Nsl1 suggests the human and fly Mis12 complexes are similar in length. The relative position of KMN subunits along the proximal–distal axes of the assembled kinetochore reflects the sequence of recruitment of the two main subcomplexes: the Mis12 complex is first assembled onto the centromere and then the Ndc80 complex is recruited so as to position the major microtubule-binding complex most distal to the centromere. Spc105 lies at a key position with its C-terminal part at the interface between the Mis12 and Ndc80 complexes.

Several pieces of evidence point to the importance of the C-terminal part of Spc105 for the assembly of the kinetochore super-structure. If this part is separated from its nuclear exclusion signal, it remains in the nucleus in interphase and enables Nsl1 to join Mis12 and Nnf1a/b at the centromere (figure 7*b*). This again suggests the existence of a ‘pre-kinetochore’ during interphase that is primed for the association of core structural proteins at centromeres and that only the absence of Spc105 prevents kinetochore assembly from being initiated. The details of how the accessibility and intracellular transport of Spc105 and other KMN network components are regulated in relation to cell cycle progression remain to be uncovered.

Targeting the C-terminal part of Spc105 to centrosomes enables recruitment of not only all other KMN components, but also CENP-C to these ectopic sites once the nuclear envelope has broken down. Moreover, as we have found using high-resolution microscopy, the position of the C-terminal part of Spc105 in the assembled kinetochore would potentially enable it to interact with Nsl1 and Mis12 of the Mis12 complex and Spc25/Mitch of the Ndc80 complex, and so would be in accord with its ability to recruit all components of the KMN network to an ectopic site. The carboxy-terminal part of Spc105 seems to be required and sufficient to drive Mis12 complex assembly. Spc105 is also known to bind several other kinetochore components crucial for proper chromosome segregation, including a protein phosphatase 1 family member and the checkpoint proteins, Bub1 and BubR1. Thus, it seems to be a critical regulatory molecule in controlling both the assembly of the KMN network and checkpoint activities of *Drosophila* kinetochore.

## 5. Conclusions

In order to precisely time the association of KMN network components with the centromere at mitotic entry, we have taken advantage of the synchronized nuclear division cycles of syncytial *Drosophila* embryos. In this way, we could accurately determine the kinetics of association of individual GFP-tagged KMN network components into kinetochores. This led us to study the roles of the C-terminal part of Spc105 in providing a hub for kinetochore assembly. Together, our findings lead to a model of the *Drosophila* KMN network assembly in which the intracellular transport of Spc105 plays a key role (figure 7*d*): in interphase, Mis12 and Nnf1a/b are associated with the centromere and Spc105 is actively excluded from nuclei. Upon mitotic entry, Spc105 is imported into the prophase nucleus, where its C-terminal part promotes rearrangement of the Mis12–Nnf1a/b–centromere interface and recruitment of the Nsl1 protein to the mitotic Mis12 complex. This provides a platform that is ready to recruit the Ndc80 complex and other kinetochore components upon NEB. This points to the importance of the previously underestimated role of cellular compartmentalization and regulation of the physical accessibility of individual KMN proteins for kinetochore assembly.

## 6. Material and methods

### 6.1. cDNAs and DNA constructs

The cDNA for *D. melanogaster Spc105* (also known as *Spc105R* or *dmSpc105R*) was amplified from total cDNA (synthesized on RNA isolated from Dmel-2 cells) according to the Superscript Reverse Transcriptase Kit (Invitrogen) protocol. Translation of the coding gave a 1959 amino acid-long product that differed from the sequence on FlyBase in three amino acids: 963 was K (not Q), 1195 was S (not T) and 1870 was A (not D). *Mitch/Spc25* cDNA (LD37196) was obtained from the Drosophila Genomics Resource Center. Constructs used in this study were generated by the Gateway Cloning System (Invitrogen). The full *Spc105*, *Mitch*, the C-terminal part of *Spc105* (1173–1959 aa) and the *Spc105* KTBD region (aa 1566–1959) were PCR amplified from cDNA templates. Products of PCR reactions were integrated by BP reactions into pDONR221 and sequenced. C- or N-terminal entry clones were combined in LR reactions with destination vectors for UASp promoter-driven expression of EGFP fusion proteins in flies [18] or with destination vectors for Actin5 promoter-driven expression of EGFP fusion proteins in cells [38].

### 6.2. Culturing and fluorescence imaging of cells

Dmel-2 cells (Invitrogen) were grown in Express Five SFM (Invitrogen) media according to standard procedures. FuGENE HD (Roche) reagent was used for transient transfection. For the purpose of imaging, cells were fixed with formaldehyde on coverslips 12 h after transfection and stained as described previously [16]. Images were taken on a Zeiss Axiovert 200 M microscope (objective 100×/1.4) with a Cool- SNAP HQ camera (Photometrics) using MAG Biosystems Software—Metamorph (Molecular Devices).

RNAi experiments were performed as described previously [16]; dsRNA-targeting kanamycin-resistance gene was used as negative control in all experiments.

### 6.3. Super-resolution optical imaging of the metaphase kinetochore

For three-dimensional SI microscopy studies (3D-SIM [39]), D-mel2 cells were fixed and immunostained as described previously [16]. We used primary antibodies directed against the following proteins: CID [16], Rod [40], Mis12, Ndc80, Nsl1 [18], Nnf1a/b and Spc105 [22], diluted as indicated. Secondary antibodies conjugated with Alexa Fluor 488 or 594 (Invitrogen) were applied in 1 : 500 dilution and cells were mounted in VectaShield media (Vector Laboratories). DNA was counterstained with DAPI. Imaging was performed using DeltaVision OMX 3D-SIM System (Applied Precision). All data capture used an Olympus 100×/1.4 oil objective, 405, 488 and 593 nm laser illumination, and standard excitation and emission filter sets. 3D-SIM images were sectioned using 125 nm Z-step size. Raw three-phase images were rendered and reconstructed in three dimensions by softWoRx 4.5.0 (Applied Precision) software. The acquired three-dimensional image stacks provided 118 nm pixel^−1^ resolution along *x-* and *y*-axes, and 125 nm steps along the *z*-axis (electronic supplementary material, figure S5*a* and video S7). Therefore, the resolution of the raw dataset is the highest among published light microscopy raw datasets about the structure of the kinetochore [24,26,27]. Objects of our measurements were bioriented sister kinetochore pairs, aligned to the metaphase plate in parallel orientation with the longitudinal spindle axis. In kinetochores, fitting the above criteria, we measured the distance of kinetochore components from CID by line scan analysis (electronic supplementary material, figure S5*b*). We defined the distance between CID and the kinetochore protein as the distance of maximal signal intensities along the intercentromeric axis (electronic supplementary material, figure S5*c*,*d*). The accuracy of our measurements was defined by measuring the distance between single kinetochore proteins and the GFP tag, which was fused to the same protein (see one example in electronic supplementary material, figure S5*f*,*h*).

### 6.4. Live imaging of embryos and quantification of kinetochore recruitment

Transgenic flies, expressing EGFP fused to Mitch, Spc105 or Spc105-C proteins, were generated by standard P-element-mediated germ line transformation. *UASp-Mis12::EGFP*, *UASp-Nsl1::EGFP* and *UASp-Nuf2::EGFP* lines used in this study were previously published [18]. To express fusion proteins in the early embryo, we used the *α*-*tubulin*^*4*^–*GAL4–VP16* driver [41]. Single copies of the driver and the UASp transgenes were combined in female flies by standard crosses. The animals were raised on standard food and kept at 25°C. Eggs aged 0–1 h were collected from fertilized females. Dechorionated embryos were air dried and covered by halocarbon oil. Subsequently, rhodamine-conjugated bovine tubulin (Cytoskeleton) was microinjected into the syncytial embryos. Microinjection and subsequent live imaging were performed on a Zeiss Axiovert 200 M microscope equipped with InjectMan (Eppendorf) micromanipulator and CellTram Oil (Eppendorf) manual microinjector. Distributions of the EGFP and rhodamine fluorochromes in the embryo cortex were detected through mitoses 11–13 as follows: Z-stacks of 15–18 non-saturated images (stack step 0.5 µm) were acquired every 18 s on the green and red channels (488 and 568 nm laser lines) of a Perkin Elmer UltraVIEW RS III spinning disc confocal system with a Zeiss 63×/1.4 oil immersion objective. Images were captured by a Hamamatsu 1394 ORCA-ERA camera and processed with Volocity 5.5 (Perkin Elmer) software. Embryos were kept at 25°C during the experiment. According to our preliminary investigations, spatial and temporal features of mitosis 12 make this cleavage cycle the best for quantitative comparison of recruitment dynamics of EGFP-tagged kinetochore proteins. Therefore, we restricted our quantitative analysis to mitosis 12. Zero point of time series was determined for each investigated nucleus independently by observing AO on the green channel. We defined as zero time point the first image where sister kinetochores were separated. To visualize and measure the EGFP signal intensities at the kinetochores, we made maximum signal intensity projection of 13 Z-stacks (comprising the whole volume of a nucleus). Subsequently, the EGFP signal accumulation at the kinetochore was quantified in single nuclei as described in electronic supplementary material, figures S1 and S2. Post-acquisition image processing and quantifications were performed with ImageJ software.

### 6.5. Immunostaining of fixed *Drosophila* embryos

Embryos aged 0–2 h were collected, dechorionated, fixed, removed from vitelline membrane, rehydrated and blocked for immunostaining exactly as described previously [42]. DM1A mouse monoclonal anti-α-tubulin (1 : 1000) and anti-centrosomin (1 : 200) were used for immunostaining overnight at 4°C. The primary antibodies were applied in 1 per cent BSA in PBST. After several rinses in PBST, the embryos were incubated in secondary antibodies for 2 h at room temperature. AlexaFlour488 and 564 secondary antibodies (Molecular Probes) were used in 1 : 500 dilutions. To detect DNA, we stained the embryos with DAPI (1 : 1000) in the first washing step of three (each 20 min) after incubation with secondary antibodies. The embryos were mounted in Aqua PolyMount (Polysciences Inc). Optical sections were generated on Zeiss LSM 510 with 63×/1.4 oil lens.

### 6.6. Ectopic localization of KTBD of Spc105

Stable cell lines were generated using Dmel-2 cells, FugeneHD transfection reagent (Promega) and the pCoBlast vector (Invitrogen) for the selection with Blasticidine (PAA) as described previously [43]. The cDNA coding for the C-terminal 394 amino acids of Spc105 was PCR amplified and fused with the centrosomal-targeting domain of Plk4/SAK exactly as described previously [22]. ectKTBD-GFP expression was induced overnight; cells were plated on coverslips, fixed with 4 per cent formaldehyde and stained with antibodies as described above. RNAi experiments were performed as described by Przewloka *et al*. [16].

## Supplementary Material

ESM_1
