# Supplementary material for: Spatiotemporal dynamics of Spc105 regulates the assembly of the Drosophila kinetochore
Source: Open Biol. 2012 Feb;2(2):110032. doi: 10.1098/rsob.110032 (PMC3352094; doi:10.1098/rsob.110032)
Supplement: ESM_1 [file rsob110032-s1.pdf]

Supplementary Information

Supplementary Kinetic Analysis

The curves presented in Figure 2A suggested co-dependent recruitment and assembly of the mitotic Mis12 complex and the nuclear accumulation of Spc105 and the sequential, Mis12/Spc105 dependent kinetochore recruitment of the Ndc80 complex. The reversible binding of a molecule of interest (Y) to another molecule already at the kinetochore (X) can be described by the following differential-equation:

$$\frac{d\,XY}{dt} = k_{ass} \cdot X \cdot Y_{free} - k_{diss} \cdot XY = k_{ass}^{'} \cdot (X_T - XY) - k_{diss} \cdot XY \qquad (1)$$

where XY is the level of the XY complex at kinetochore which is measured by fluorescently tagged Y protein. In this case we describe a two-step, sequential binding process; in the first step X is the Mis12 complex and Spc105 is Y and in the second step, X and Y are the Mis12 and Ndc80 complexes, respectively. The right hand-side of the equation can be rearranged based on the following considerations. First, the level of X not bound to Y at a kinetochore can be expressed as the difference between total X at the kinetochore (X_T) minus the complex: $X=X_t$-XY. Secondly, assuming the free pool of Y (Y_{free}) is large and constant, it was grouped together with the association rate constant thus giving a first order association rate constant (k_{ass}' = $k_{ass} \cdot Y_{free}$). Because of sequential binding of molecules to the kinetochore, X_T is not constant, but rather it is a monotonously increasing function of time. Therefore two extreme cases can be distinguished depending on the relative rate of change in X_T increase and of Y binding to X. If Y binding to X is fast compared to the change in level of X_T, then d XY/dt =0 and Equation 1 reduces to:

$$XY = \frac{X_T}{1 + \frac{k_{diss}}{k_{ass}^{'}}} \qquad (2)$$

In this case, the level of Y at the kinetochore (XY) will be proportional to the total level of X (X_T). However, if Y binding is relatively slow compared to the change in total X at a kinetochore (X_T) then for small XY values:

$$\frac{d\ XY}{dt} \cong k_{ass}^{'} \cdot X_T - k_{diss} \cdot XY \qquad (3)$$

i.e. the rate of Y accumulation at kinetochores will be proportional to X_T.

Based on these considerations, we analyzed the kinetics of kinetochore protein recruitment (Fig.2a) from interphase until anaphase (time=0). By plotting the level of the Mis12 complex component, Nsl1, as a function of Spc105 levels, we found a linear relationship according to Equation 2 (Fig. 2c). The slope and the intercept of the regression line were not significantly different form one and zero thus validating this Equation. This suggests that the Mis12 complex and Spc105 bind to each other very fast and that $k_{diss} << k_{ass}$'.

In contrast, Ndc80 complex level (assessed as the mean value for its two equimolar components (Nuf2 and Mitch) at kinetochores does not show linear correlation with Mis12 complex (not shown). However, when we calculated the rate of accumulation of Ndc80 complex at kinetochores (d XY/dt), the slope of the blue curves from Fig.2a, and the level of Nsl1 (X_T) as a function of time, we found a strong correlation (Fig.2d). This suggests that Ndc80 binding to Mis12 is a slow and reversible process. The dissociation and the apparent first order association rate constants of their binding was estimated after rearranging Equation 3 by dividing both sides with XY:

$$\frac{1}{XY}\frac{d\ XY}{dt} \cong k_{ass}^{'} \cdot \frac{X_T}{XY} - k_{diss} \qquad (4)$$

By plotting the specific rate of Ndc80 accumulation ($1/XY \cdot dXY/dt$) as a function of Mis12 Ndc80 ratio gives straight with a slope and intercept corresponding to k_{ass}' and k_{diss} values of $4.6 \cdot 10^{-3}$ and $1.8 \cdot 10^{-3}$ sec^{-1} (Fig.2e).

Supplementary Figures

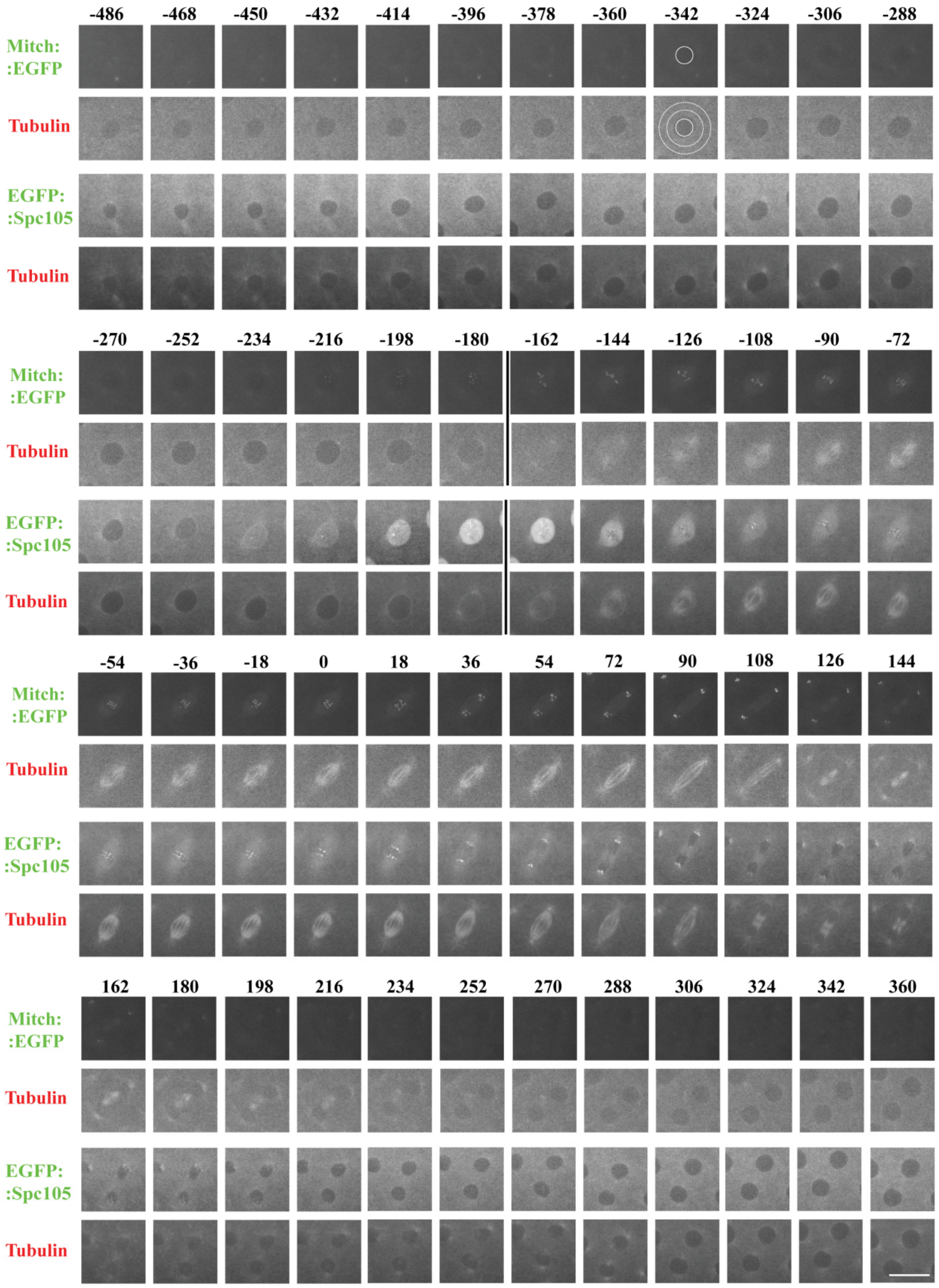

Supplementary Figure S1

Figure S1. Time frames of single nuclei in cleavage division cycle 12 from Mitch::EGFP and EGFP::Spc105 embryos. Maximum intensity projected z stacks were aligned in time to anaphase onset. The first time frame after anaphase onset was defined as 0 sec time point. In every 18 sec images were captured on two channels to detect EGFP fusion protein and rhodamine-conjugated tubulin. Circle at -342 sec shows the ROI (region of interest) used on green channel in every time point to quantify EGFP signal accumulation at the kinetochores. ROIs with exactly the same coordinates were used on the red channel to define average rhodamine-tubulin signal intensity from interphase to anaphase onset (AO) in the nucleus until nuclear envelope break down (NEB) and around the chromosomes from NEB. In the same time interval average rhodamine-tubulin signal intensity in the cytoplasm was measured in the area between the two dotted line circles shown at -342 sec. After black bars between -180 sec and -162 sec the average rhodamine-tubulin signal intensity in the nucleus turns to be higher than in the cytoplasm. Scale bar represents 10 μm.

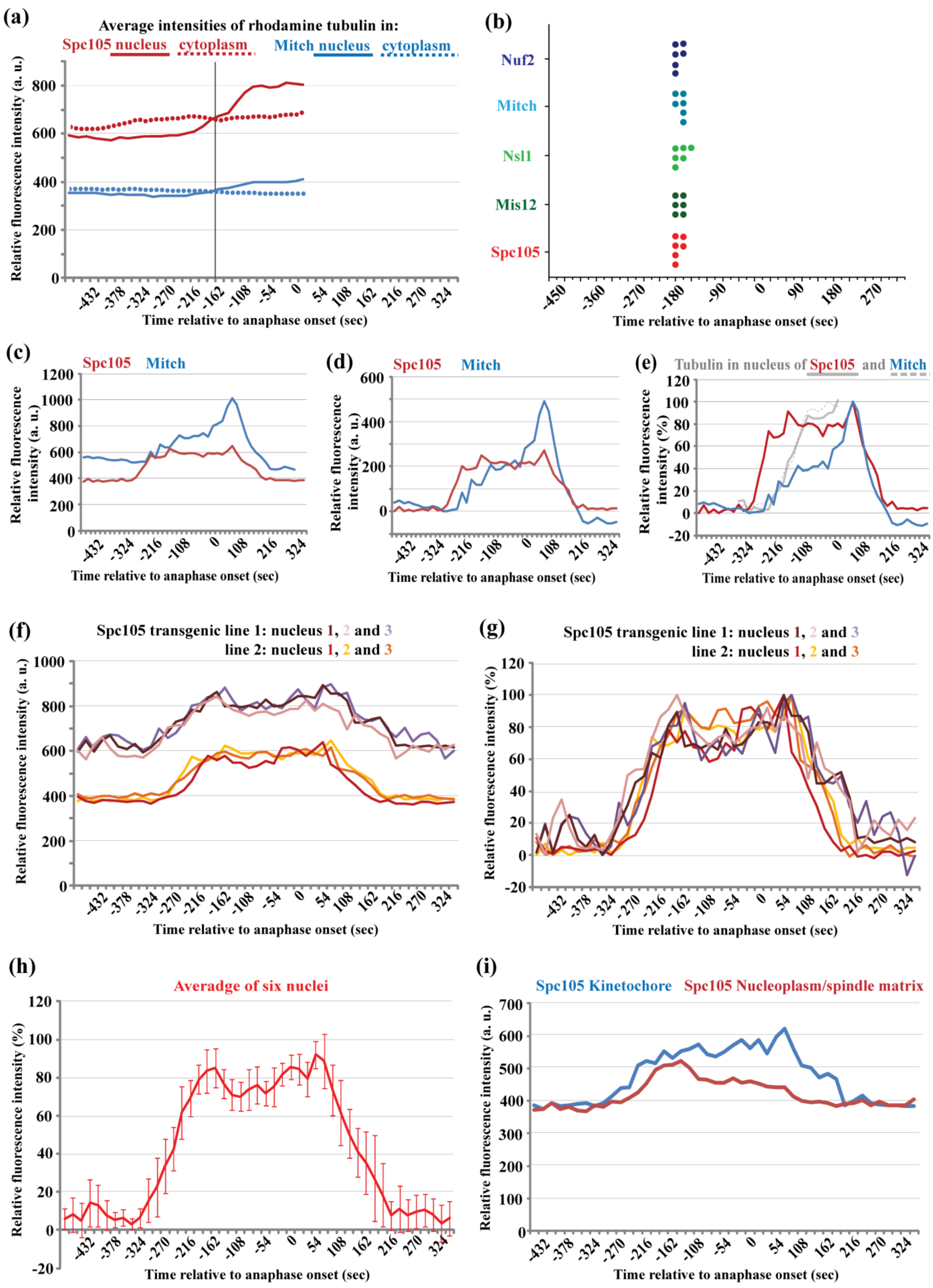

Supplementary Figure S2

Figure S2. Quantification of kinetochore assembly in mitosis 12.

(a) To validate the alignment of data sets from different nuclei in time, we measured the kinetics of rhodamine tubulin accumulation in the nuclei. Average signal intensities in the nucleus and in the cytoplasm were plotted as function of time and date series from different nuclei were aligned to AO as in Supplementary Figure S1. Data of the two nuclei from Figure S2 (Mitch::EGFP in blue and EGFP::Spc105 in red as in each panel of this figure) are represented. Continuous lines represent the average signal intensities in the nuclei and around the chromosomes. Dotted lines represent the average signal intensities in the cytoplasm. Black bar defines the border between time frames with less and more nuclear versus cytoplasmic average signal intensities (identical to Supplementary Figure S1).

(b) Nuclear envelope breakdown occurs with uniform timing: Dot plot diagram presenting the distribution of time points where average rhodamine tubulin signal intensities in the nuclei becomes to be higher than in the cytoplasm (as in Supplementary Figure S1). Time points are from data sets of 6 nuclei per fusion constructs.

(c) Maximal signal intensities in the green channel in the ROI defined in Figure S1 plotted as function of time.

(d) Maximal signal intensities form panel (b) normalised to the minimum value in interphase to prophase 12 period (interval -486 sec to -180 sec).

(e) The same data presented with a relative scale for signal intensities where 0 is the minimum value from interval -486 sec to -180 sec, and 1 is the highest value in the entire data sequence. Gray curves represent the average intensities of rhodamine tubulin in the nuclei from panel (a). Data were transformed to the relative scale similar to kinetochore signal data (continuous line Mitch, dotted line Spc105).

(f) Reproducibility of Spc105 import between embryos: Maximal signal intensities in the green channel at the kinetochores of 3-3 nuclei of two EGFP::Spc105 embryos plotted as function of time.

(g) Maximum intensity values from panel (b) presented with a relative scale of signal intensity. Relative scale was defined for each nucleus separated as above in (e).

(h) The average of values from panel (c). Error bar represents standard deviation.

(i) Quantitative comparison of Spc105 kinetochore recruitment and accumulation into the nucleus/spindle matrix in mitosis 12. Maximal signal intensity in the nucleus and at the chromosomes after NEB represents the accumulation of GFP::Spc105 at the kinetochore (blue line). Maximum signal intensities in the nucleoplasm before NEB and in the spindle matrix after NEB represent the dynamics of Spc105 nuclear import (red line). Data indicate no delay in kinetochore accumulation after nuclear import.

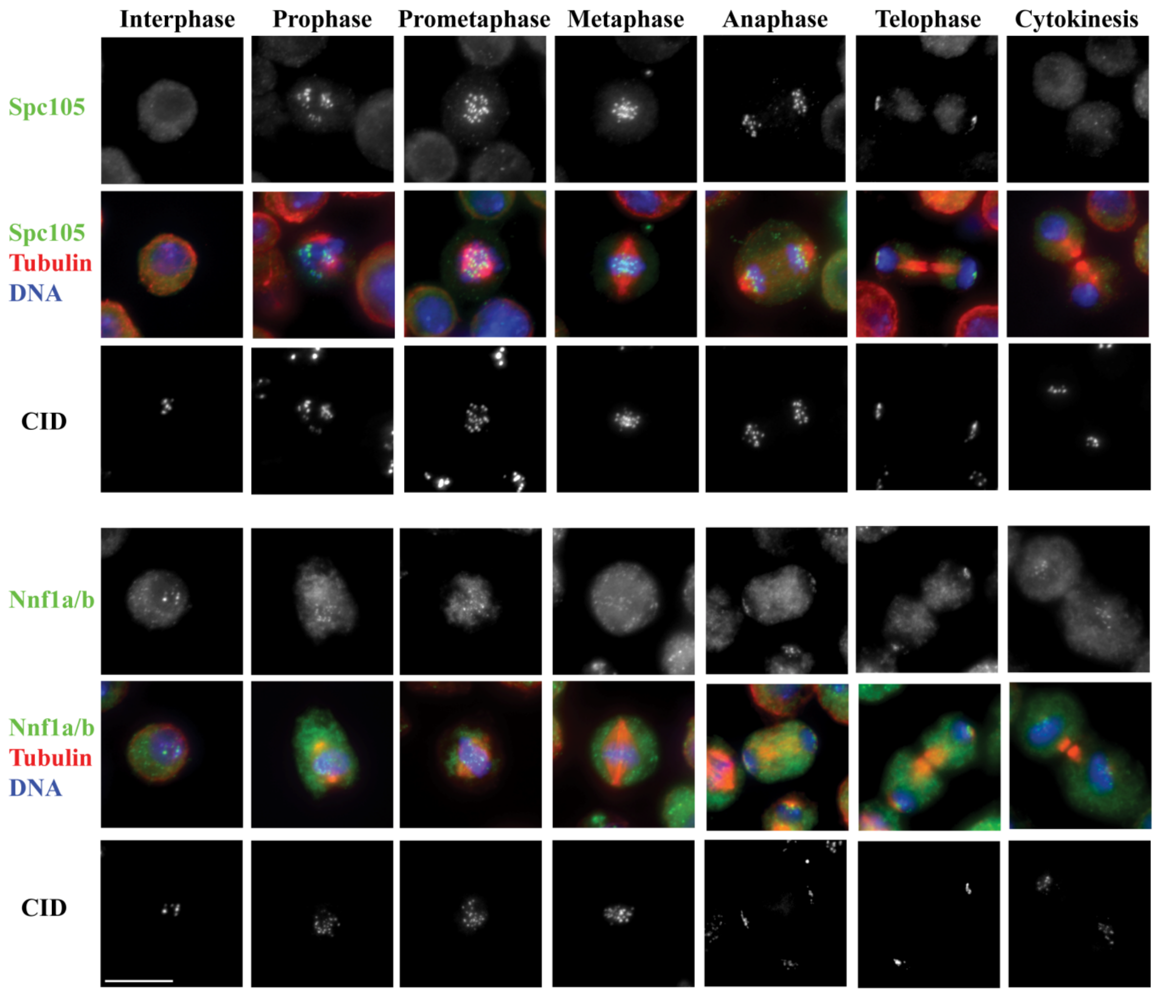

Supplementary Figure S3

Figure S3. Localization of Spc105 and Nnf1a/b throughout the cell cycle. Specificity of polyclonal antibodies against Spc105 and Nnf1a/b were determined by Western Blotting before (Przewloka et al., 2011). Fixed D-mel2 cells were stained for Spc105 or Nnf1a/b (in green), tubulin (in red), DNA (in blue) and CID (in greyscale, not merged). Spc105 localizes to the kinetochore in mitosis. Nnf1a/b is at the centromere throughout the entire cell cycle. Nnf1a/b signal intensity at the centromere is the weakest in metaphase.

Scale bar represents 10 µm.

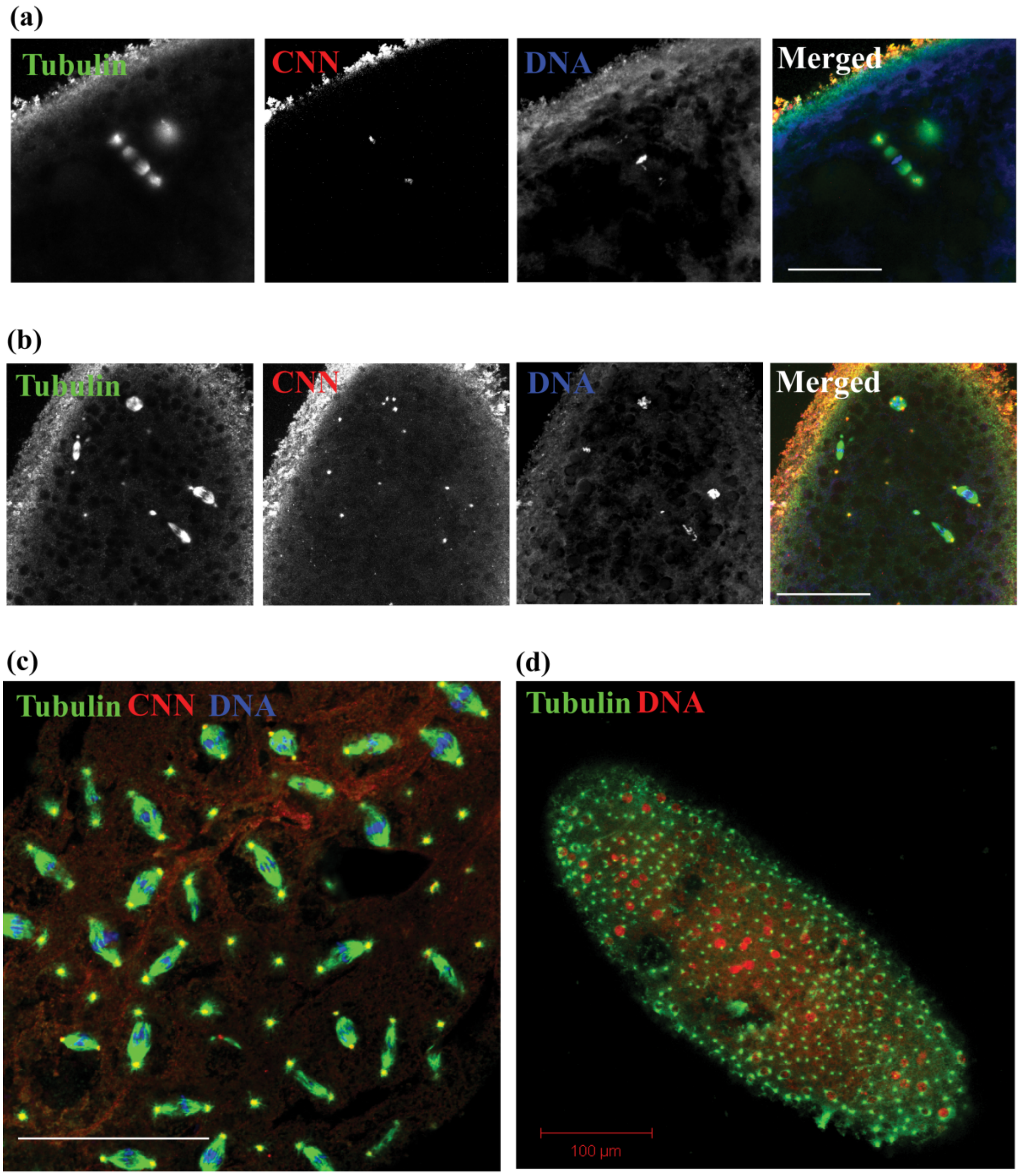

Supplementary Figure S4

Figure S4. Spc105-C over-expression caused mitotic defects in the syncytial embryo.

Spc105-C over expression causes developmental arrest and lethality before cellularization of the embryo.

(a) Elongated anaphase spindle with non segregating chromosomes and with DNA fragments in the spindle poles are characteristic for early arresting embryos (vast majority of the embryos).

(b) Characteristic phenotypes in the embryos which completed the first mitotic divisions: centrosome detachment in mitosis and spindles collapse.

(c) Terminally arrested phenotype - enlarged cortical region

(d) Terminally arrested phenotype in full view of an embryo with late stage developmental arrest. Most embryos arrested before mitosis 10 when Spc105-C expression was driven by mat-tub-Gal4. When expression was weaker, under nanos-Gal4 control, embryos mainly arrested in mitosis 10-13 (Video 6 is also of such an embryo).

Scale bar 20 µm for (a), (b) and (c), and 100 µm for (d).

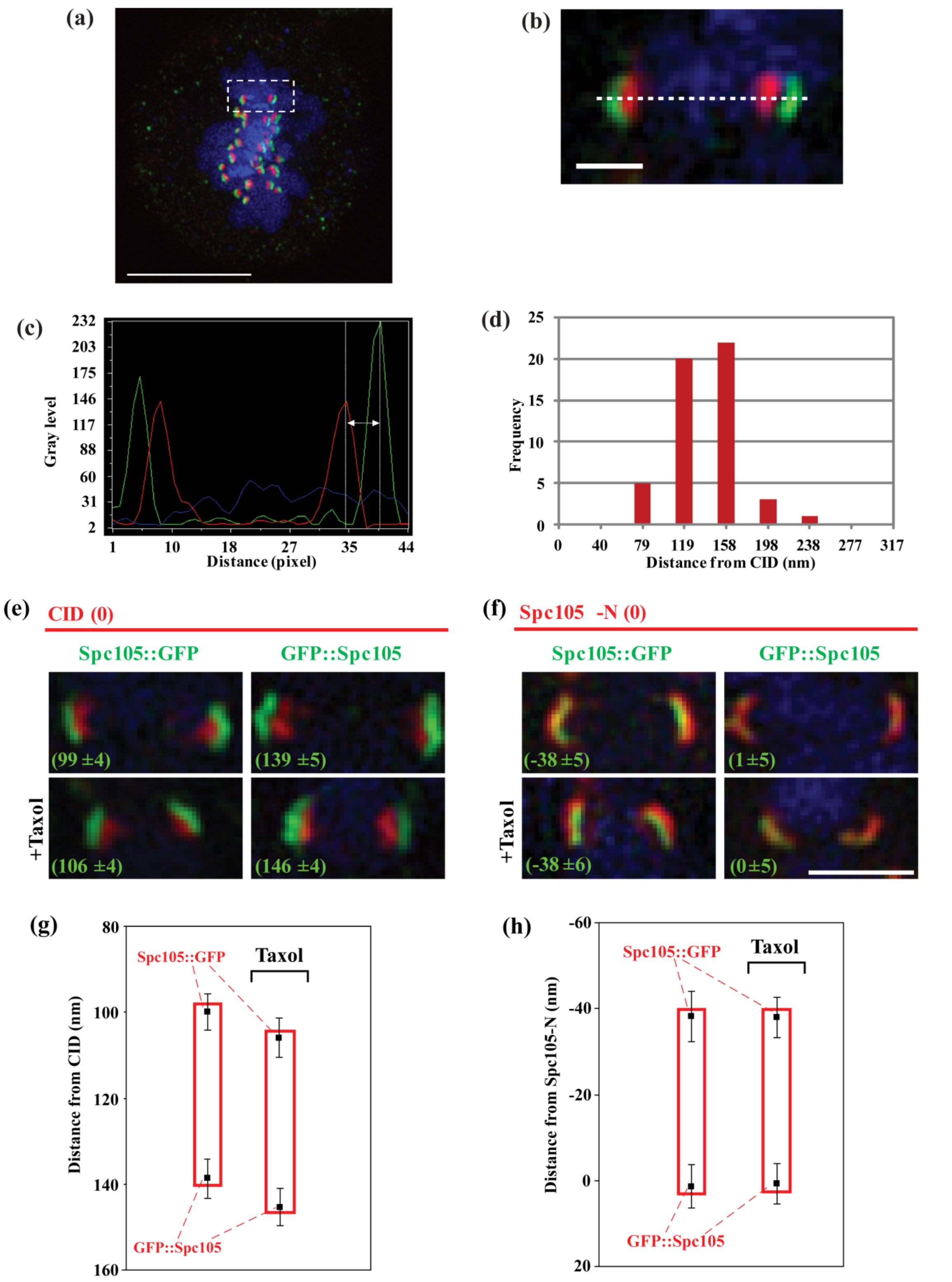

Supplementary Figure S5

Figure S5. Quantification of intrakinetochore distances and the response to loss of tension.

(a) Maximum intensity projection of 38 z-sections, acquired by the OMX microscope in SI mode about a D-mel2 cell in metaphase. The equatorial plane of the mitotic spindle is perpendicular to the focal plane of the objective. CID in red, Spc105-N in green and DNA in blue. Scale bar represents 5 µm, z-step were 125 nm.

(b) Maximum intensity projection of 3 z-slides from the marked box area of (a). The middle slide has the highest signal intensities both for CID and Spc105-N. Scale bar represents 500 nm.

(c) Line scans of signal intensities on three channels along the scattered line shown in (b). Distance between maxima of CID and Spc105-N signal intensities in the kinetochores are highlighted by double-ended arrows.

(d) Distribution of distance values measured between maxima of CID and Spc105-N signal intensities in kinetochores of 25 sister kinetochore pairs. The 25 sister kinetochores were bioriented, parallel to the focal plane, with intercentromeric distances in a range of 800-1250 nm.

(e,f) Bioriented sister kinetochore pairs in D-mel2 cells, expressing Spc105 fused GFP, after treatment with DMSO (upper panels), or 50 nM Taxol. GFP in green, CID in red, DNA in blue, CID in red for (e), and Spc105-N in red for (f). Scale bar represents 1000 nm.

(g). Shift in position of Spc105 ends from CID (*n*=50) in Taxol treated cells.

(h) Axial position of GFP tags relative to Spc105-N epitope along the sister kinetochore axis in Taxol treated cells. Positive values are outward (toward the spindle microtubules), negative values are inward (toward centromeric chromatin). *n*=50, error bars represents SEM for (g) and (h).

Supplementary movies

Video 1

Cleavage division 12 and 13 in a Mitch::EGFP embryo. Frames were taken in every 18 sec. Zero time point is the first frame after anaphase onset in mitosis 12. Mitch::EGFP in green, tubulin in red. Frames were taken in every 18 sec. Time span 2124 sec.
Scale bar 10 μm.

Video 2

Cleavage division 12 and 13 in a EGFP::Spc105 embryo. Frames were taken in every 18 sec. Zero time point is the first frame after anaphase onset in mitosis 12. EGFP::Spc105 in green, tubulin in red. Frames were taken in every 18 sec. Time span 2088 sec.
Scale bar 10 μm.

Video 3

EGFP::Spc105 nuclear import and centromeric accumulation in prophase of cortical cleavage division 12. Signal distribution in a single focal plane of the nucleus (left panel), also shown as surface intensity plot in the selected area of the nucleus (right panel).
Frames were taken in every 30 sec. Time span, 480 sec.

Video 4

Mitosis 14 in a EGFP::Spc105 embryo. Frames were taken in every 30 sec.
Time span 1290 sec.

Video 5

Cortical cleavage divisions in EGFP::Spc105 embryo. Frames were taken in every 18 sec. Time span 936 sec.

Video 6

Cortical cleavage divisions in EGFP::Spc105-C embryo. Frames were taken in every 18 sec. Time span 1386 sec.

Video 7 Bioriented sister kinetochores in a metaphase D-mel2 cell. 3D volume of the z-stack about the metaphase D-mel2 cell presented in Supplementary Figure S5A. CID in red, Spc105-N in green and DNA in blue.

Supplementary Information Reference

Przewloka, M.R., Z. Venkei, V.M. Bolanos-Garcia, J. Debski, M. Dadlez, and D.M. Glover. 2011. CENP-C Is a Structural Platform for Kinetochore Assembly. *Curr Biol*. 21:399-405.
